# A Robust Nanoparticle Platform for RNA Interference in Macrophages to Suppress Tumor Cell Migration

**DOI:** 10.3389/fphar.2018.01465

**Published:** 2018-12-14

**Authors:** Shi Liang, Junmeng Zheng, Wei Wu, Quan Li, Phei Er Saw, Jianing Chen, Xiaoding Xu, Herui Yao, Yandan Yao

**Affiliations:** ^1^Guangdong Provincial Key Laboratory of Malignant Tumor Epigenetics and Gene Regulation, Medical Research Center, Sun Yat-sen Memorial Hospital, Sun Yat-sen University, Guangzhou, China; ^2^RNA Biomedical Institute, Sun Yat-sen Memorial Hospital, Sun Yat-sen University, Guangzhou, China; ^3^Breast Tumor Center, Sun Yat-sen Memorial Hospital, Sun Yat-sen University, Guangzhou, China

**Keywords:** macrophages, RNAi, nanoparticle, cancer therapy, siRNA delivery

## Abstract

Macrophages are one of the most abundant immune cells in the solid tumor and their increased density is associated with the specific pathological features of cancers, including invasiveness, metastasis, immunosuppression, neovascularization, and poor response to therapy. Therefore, reprogramming macrophage behavior is emerging as a promising therapeutic modality for cancer treatment. RNA interference (RNAi) technology is one of the powerful strategies for the regulation of macrophage activities by silencing specific genes. However, as polyanionic biomacromolecules, RNAi therapeutics such as small interfering RNA (siRNA) cannot readily cross cell membrane and thus specific delivery vehicles are required to facilitate the cytosolic siRNA delivery. Herein, we developed a robust nanoparticle (NP) platform for efficient siRNA delivery and gene silencing in macrophages. This NP platform is composed of biodegradable poly (ethylene glycol)-*b*-poly (*𝜀*-caprolactone) (PEG-*b*-PCL), poly (*𝜀*-caprolactone)-*b*-poly (2-aminoethyl ethylene phosphate) (PCL-*b*-PPEEA), and PCL homopolymer. We chose CC-chemokine ligand 18 (CCL-18) as a proof of concept therapeutic target and our results demonstrate that the CCL-18 silencing in macrophages can significantly inhibit the migration of breast cancer cells. The successful regulation of the macrophage behavior demonstrated herein shows great potential as an effective strategy for cancer therapy.

## Introduction

Macrophages are important cells of immune system with two major phenotypes, i.e., pro-inflammatory phenotype (M1) and anti-inflammatory phenotype (M2) (Mantovani et al., 2008; Noy and Pollard, 2014; Ostuni et al., 2015). In solid tumors, tumor-associated macrophages (TAMs) are one of the most abundant cell types (up to 50% of the tumor mass) and are present at all stages of tumor progression (Caillou et al., 2011; Ginhoux et al., 2015; Parayath et al., 2018). Numerous clinical and epidemiological studies have demonstrated that TAMs are primary M2-like macrophages (Gordon and Martinez, 2010; Sica and Mantovani, 2012; Bronte and Murray, 2015) and their increased density is associated with the specific pathological features of cancers, including invasiveness, metastasis, immunosuppression, neovascularization, and poor response to therapy (Qian and Pollard, 2010; McAllister and Weinberg, 2014; Bronte and Murray, 2015). Therefore, macrophages represent an important therapeutic target and strategies that can effectively regulate undesirable macrophage activities are always pursued for future cancer therapy.

One of the promising strategies for the regulation of macrophage activities is using RNA interference (RNAi) technology to silence specific genes (Aouadi et al., 2009; Kortylewski et al., 2009; Yu et al., 2013). Since its discovery in 1998, RNAi technology has demonstrated significant potential for disease treatment by silencing the expression of target gene(s), especially those encoding “undruggable” proteins (Melnikova, 2007; Burnett and Rossi, 2012; Kanasty et al., 2013; Zuckerman and Davis, 2015). The key challenge is the safe and effective delivery of RNAi therapeutics such as small interfering RNA (siRNA) to aberrant macrophages (e.g., TAMs). Due to its polyanionic and macromolecular characteristics, naked siRNA cannot readily cross the cell membrane and thus requires specific delivery vehicles to facilitate its intracellular uptake and cytosolic delivery for bioactivity (Whitehead et al., 2009; Zhang et al., 2012; Shi et al., 2017; Xu et al., 2017b). Over the past decade, nanoparticles (NPs), which present the advantage of preferential and selective accumulation at tumor sites via the enhanced permeation and retention (EPR) effect, have been widely used for cancer treatment (Chen et al., 2017; Zeng et al., 2017; Liu et al., 2018; Xiao et al., 2018). Up to now, numerous innovative NPs have been developed to enhance the siRNA delivery efficacy (Tseng et al., 2009; Xu et al., 2016, 2017a; Cheng et al., 2017; Saw et al., 2018). However, a substantial number of these NPs are designed to directly target tumor cells. At present, modest effort has been made to develop RNAi NPs for the modulation of undesirable macrophage activities.

**FIGURE 1:**
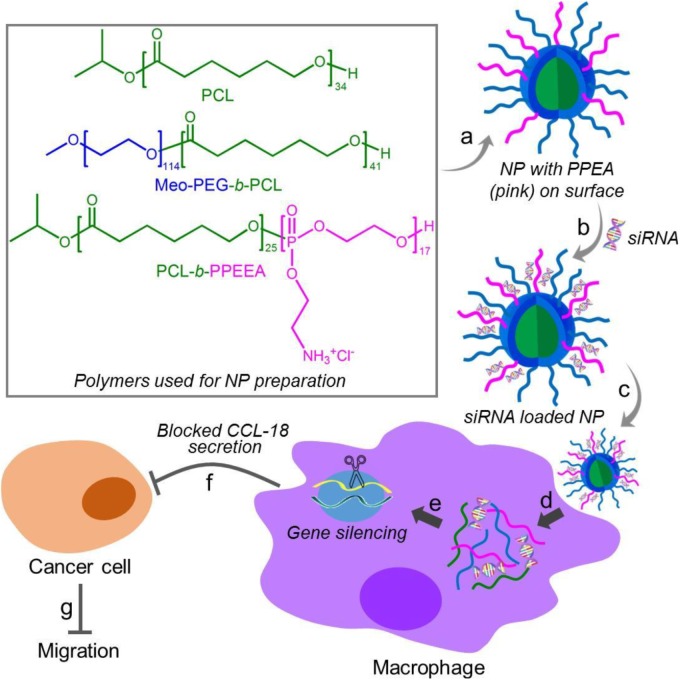
Chemical structures of the polymers (PCL, Meo-PEG-*b*-PCL, and PCL-*b*-PPEEA) and schematic illustration of the self-assembly of the polymers into nanoparticles (NPs) for small interfering RNA (siRNA) delivery and CCL-18 silencing in macrophages to inhibit breast cancer migration. The amphiphilic Meo-PEG-*b*-PCL and PCL-*b*-PPEEA can spontaneously self-assemble into NPs with hydrophobic PCL chains embedded in the cores and hydrophilic PEG and PPEEA chains positioned on the surface **(a)**. After siCCL-18 loading via the electrostatic interaction **(b)** and then internalized by macrophages **(c,d)**, the siCCL-18 can knock down CCL-18 expression **(e)** and thus CCL-18 secretion from the macrophages would be blocked **(f)**, leading to the inhibition of tumor migration **(g)**.

Herein, we developed a robust RNAi NP platform for the efficient regulation of macrophage activities. This NP platform is composed of biodegradable poly (*𝜀*-caprolactone)-*b*-poly (2-aminoethyl ethylene phosphate) (PCL-*b*-PPEEA), poly (ethylene glycol)-*b*-poly (*𝜀*-caprolactone) (PEG-*b*-PCL), and PCL homopolymer (Figure 1). Through optimizing the NP size by adjusting the formulation, we demonstrated that larger size NPs can deliver siRNA and silence target gene in macrophages with higher efficacy. As a proof-of-concept, we chose CC-chemokine ligand 18 (CCL-18) as a therapeutic target and evaluated the influence of CCL-18 silencing on the macrophage activities. CCL-18 is a key factor secreted by TAMs to induce cancer cell epithelial-mesenchymal transition (EMT), enhance breast cancer metastasis, and reduce patient survival (Chen et al., 2011; Su et al., 2014; Nie et al., 2017). Our results show that the optimal NP platform can efficiently silence the CCL-18 expression in macrophages, leading to significant inhibition of breast cancer cell migration (Figure 1).

## Materials and Methods

### Materials

Methoxyl-poly (ethylene glycol) (Meo-PEG_114_-OH, *M_n_* = 5000), phorbol myristate acetate (PMA), *N*-2-hydroxyethylpiperazine-*N*′-2-ethanesulfonic acid buffered saline (HEPES), stannous octoate [Sn (Oct)_2_], RNase A, and heparin sulfate were acquired from Sigma-Aldrich and used directly. *𝜀*-Caprolactone (CL) was provided by Sigma-Aldrich and distilled before use. The poly (*𝜀*-caprolactone) homopolymer with 34 repeating units (PCL_34_) and polydispersity of 1.21 was synthesized according to the previous report (Wang et al., 2006). The block copolymers, methoxyl-poly (ethylene glycol)-*block*-poly (*𝜀*-caprolactone) (mPEG_114_-*b*-PCL_41_) and poly (*𝜀*-caprolactone)-*block*-poly (2-aminoethyl ethylene phosphate) (PCL_25_-*b*-PPEEA_17_), were synthesized according to our previous reports (Wang et al., 2013; Liang et al., 2015). The degree of polymerization of each repeating unit was calculated based on proton nuclear magnetic resonance (^1^HNMR) analysis. Dulbecco’s modified Eagle’s medium (DMEM), 3-[4,5-dimethylthiazol-2-yl]-2,5-diphenyltetrazoliumbromide (MTT), fetal bovine serum (FBS), and trypsin were purchased from Gibco BRL. Lipofectamine 2000 (Lipo) RNAi MAX transfection kit and DAPI were provided by Invitrogen Corp. Fluorescent dye (Cy5) labeled CCL-18 siRNA (Cy5-si*CCL-18*) and negative control siRNA (si*NC*) were acquired from Suzhou Ribo Life Science Co., The siRNA sequences are as follows: si*NC*, 5′-TTG GGA AAA TGA GTG GTT dTdT-3′ (sense) and 5′-AAC CAC TCA ACT TTT TCC CAA dTdT-3′ (antisense); si*CCL-18*, 5′-ACA AGU UGG UAC CAA CAA ATT-3′ (sense) and 5′-UUU GUU GGU ACC AAC UUG UGC -3′ (antisense). The fluorescent dye was labeled at the 5′-end of the sense strand of si*CCL-18*. All other organic solvents or reagents were analytical grade and used without further purification.

### Methods

#### Preparation and Characterizations of Nanoparticles (NPs)

The NPs with different sizes were prepared by using the classic nanoprecipitation method. The polymers, mPEG-*b*-PCL (10 mg), PCL-*b*-PPEEA (10 mg), and PCL (50 mg) were, respectively, dissolved in 1 mL of acetonitrile and methyl alcohol (v/v, 1:1). Subsequently, mPEG-*b*-PCL and PCL-*b*-PPEEA were mixed in a molar ratio of 1.5:1 and then added dropwise to 10-fold volume of deionized water which was under vigorously stirring. After stirring for another 20 min, the NP suspension was transferred into a rotary evaporator to remove the organic solvent. The final NP suspension was dispersed in deionized water at a concentration of 1 mg/mL. To adjust the NP size, different amount of PCL was mixed with the mixture of mPEG-*b*-PCL and PCL-*b*-PPEEA (molar ratio, 1.5:1) and the resulting NPs were prepared according to the same method described above. The size distribution and zeta potential of the NPs were examined by dynamic light scattering (DLS, Malvern Instruments Corporation). The morphology of the NPs was observed by transmission electron microscopy (TEM, Tecnai G^2^ Spirit BioTWIN).

#### Gel Retardation Assay

The NPs prepared above were mixed with the si*CCL-18* aqueous solution (20 mM) at different N/P ratios. After incubating at room temperature for 20 min, the formed NP/si*CCL-18* complexes were electrophoresed on a 1% agarose gel at 120 mV for 10 min in pH 8.3 Tris/borate/EDTA buffer (89 mM Tris, 89 mM boric acid, 2 mMEDTA). The siRNA bands were visualized with ethidium bromide staining under a UV transilluminator at a wavelength of 365 nm. Naked si*CCL-18* was used as control.

#### *In vitro* siRNA Release

The NP/Cy5-si*CCL-18* complexes at an N/P ratio of 10:1 were prepared according the same method described above and then suspended in pH 7.4 PBS solution at a siRNA concentration of 200 nM. Subsequently, the siRNA loaded NP suspension was transferred to a dialysis device (MWCO 100 kDa) that was immersed in pH 7.4 PBS solution at 37°C. At a predetermined interval, 5 μL of the NP suspension was withdrawn and mixed with 20-fold DMSO. The fluorescence intensity of Cy5-si*CCL-18* was determined by a microplate reader.

#### Evaluation of the Stability of NP/siRNA Complexes

The NP/si*CCL-18* complexes were prepared at an N/P ratio of 10:1 and then dispersed in DMEM containing 10% FBS at 37°C. At a predetermined interval, the size distribution of the NP/si*CCL-18* complexes was examined by DLS.

#### Cell Culture

THP-1 cells (human monocytic leukemia cell line) and human breast cancer cells (MDA-MB-231 cell line) were obtained from the American Type Culture Collection (ATCC) and incubated in DMEM medium with 10% FBS at 37°C in a humidified atmosphere containing 5% CO_2_.

#### Construction of M2-Like Macrophages

THP-1 cells were incubated in DMEM medium containing 10% FBS and PMA (10 ng/mL) at for 8 h. Subsequently, the medium was replaced by fresh DMEM medium containing interleukin-4 (IL-4, 20 ng/mL). After 48 h incubation, the cells will differentiate into M2-like macrophages and the mRNA level of CCL-18 was examined using quantitative reverse transcription real-time PCR (qRT-PCR).

#### Confocal Laser-Scanning Microscope (CLSM)

After successful construction of the M2-like macrophages, the cells (50,000 cells) were seeded in round disks and incubated in 2 mL of DMEM medium containing 10% FBS. After 24 h incubation, the medium was replaced and the NP/Cy5-si*CCL-18* complexes were added to the disks at a siRNA concentration of 200 nM. After 4 h incubation, the medium was removed and the cells were washed with pH 7.4 PBS solution thrice. Finally, the nuclei were stained with DAPI and the cells were viewed under a Carl Zeiss LSM 710 CLSM.

#### Flow Cytometry

The M2-like macrophages were seeded in six-well plate (50,000 cells per well) and incubated in 2 mL of DMEM medium containing 10% FBS. After 24 h incubation, the medium was replaced and the NP/Cy5-si*CCL-18* complexes were added at a siRNA concentration of 200 nM. After 4 h incubation, the medium was removed and the cells were washed with pH 7.4 PBS solution thrice. Finally, the cells were collected for flow cytometry analysis using a BD FACSCalibur flow cytometer.

#### Cytotoxicity

The cytotoxicity of the NPs was evaluated by using MTT assay. The M2-like macrophages were seeded in 96-well plates at 5,000 cells per well and incubated in 100 μL of DMEM medium containing 10% FBS. After 24 h incubation, the medium was removed and different amounts of the NPs suspended in the culture medium were added. After 48 h incubation, the medium was removed and the cell viability was examined using the MTT assay according to the manufacturer’s protocol.

#### CCL-18 Silencing

The M2-like macrophages were seeded in six-well plate (50,000 cells per well) and incubated in 2 mL of DMEM medium containing 10% FBS. After 24 h incubation, the medium was replaced and the NP/si*CCL-18* complexes were added at different siRNA concentrations. After 48 h incubation, the medium was removed and the cells were washed with pH 7.4 PBS solution thrice. The intracellular mRNA was isolated and the mRNA level of CCL-18 was examined using qRT-PCR.

#### Inhibition of Migration

MDA-MB-231 cells (50,000 cells) were seeded in round disks and incubated in 2 mL of DMEM medium containing 10% FBS. After 24 h incubation, the cells in the predesigned area of the disks were removed using tips. After washing the cells with PBS thrice, the cells were incubated with the conditioned medium from the macrophages, in which the CCL-18 has been silenced by the NP-180/si*CCL-18* at a siRNA concentration of 400 nM. After 48 h incubation, the medium was removed and the cells were viewed under optical microscope after washing with PBS thrice.

## Results and Discussion

### Preparation and Characterizations of Nanoplatform

**FIGURE 2:**
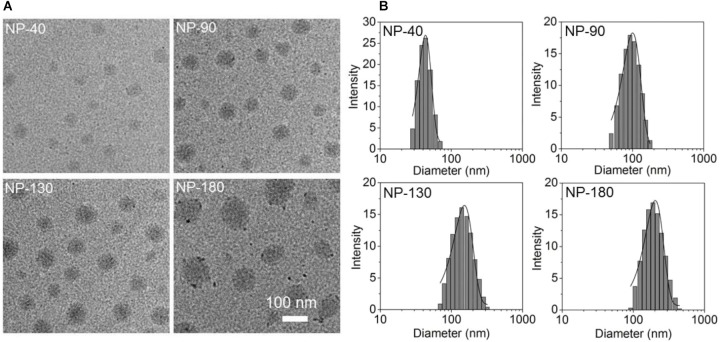
**(A)** Transmission electron microscopic (TEM) images and **(B)** size distribution of the NP-40, NP-90, NP-130, and NP-180.

Starting from the commercial available mPEG-OH and *𝜀*-CL, we employed ring-opening polymerization (ROP) to synthesize the mPEG-*b*-PCL and PCL homopolymer (Sun et al., 2008). The amphiphilic polymer PCL-*b*-PPEEA was also prepared by ROP method (Sun et al., 2008), in which the cationic PPEEA segment was used to complex siRNA via electrostatic interaction. By mixing these three polymers in acetonitrile and methyl alcohol (v/v, 50:50) followed by the addition to deionized water, well-defined NPs can be formed with spherical morphology (Figure 2A). In this self-assembly system, the amphiphilic PEG-*b*-PCL and PCL-*b*-PPEEA can spontaneously self-assemble into NPs with hydrophobic PCL chains embedded in the cores and hydrophilic PEG and PPEEA chains positioned on the surface that can, respectively, stabilize the NPs and complex negatively charged siRNA. Moreover, with the increasing feed amount of PCL homopolymer, the average size of the resulting NPs increases from ∼40 to 180 nm (Figure 2B). The possible reason is that the increased percentage of PCL homopolymer in the NPs induces the size increase of their hydrophobic inner cores (Mao et al., 2014; Liang et al., 2015). In this work, we prepared four types of NPs with the size of 40, 90, 130, and 180 nm (respectively, denoted NP-40, NP-90, NP-130, and NP-180) to evaluate their ability to deliver CCL-18 siRNA (si*CCL-18*) for gene silencing in macrophages.

**FIGURE 3:**
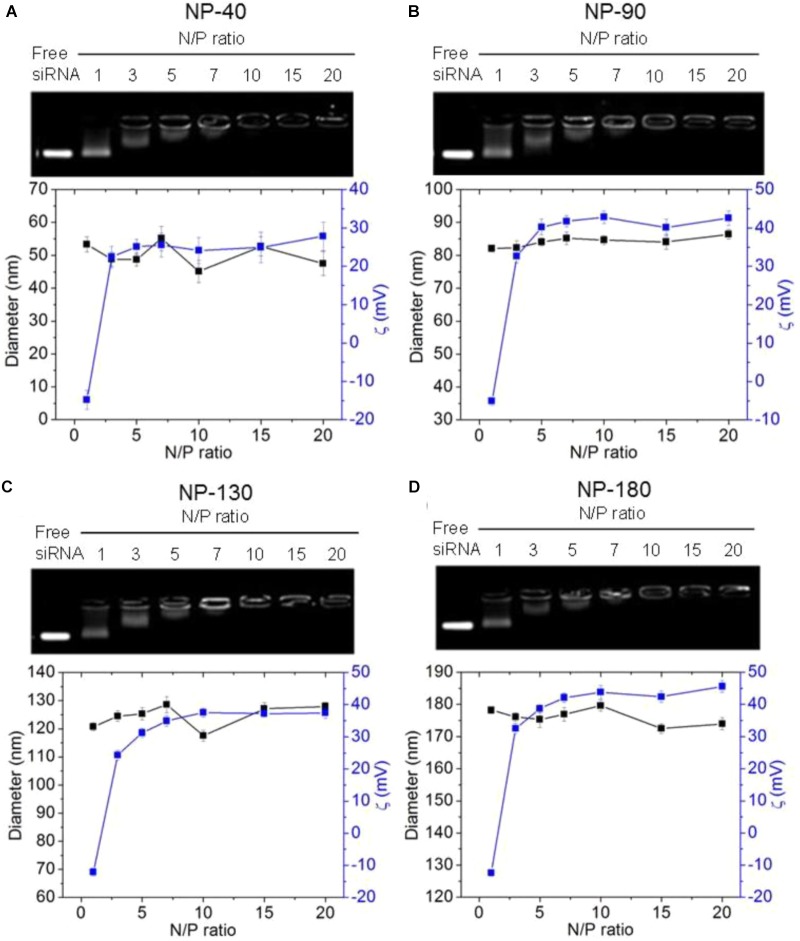
Gel retardation analysis, size, and zeta potential of the complexes formed between si*CCL-18* and the NP-40 **(A)**, NP-90 **(B)**, NP-130 **(C)**, or NP-180 **(D)**.

**FIGURE 4:**
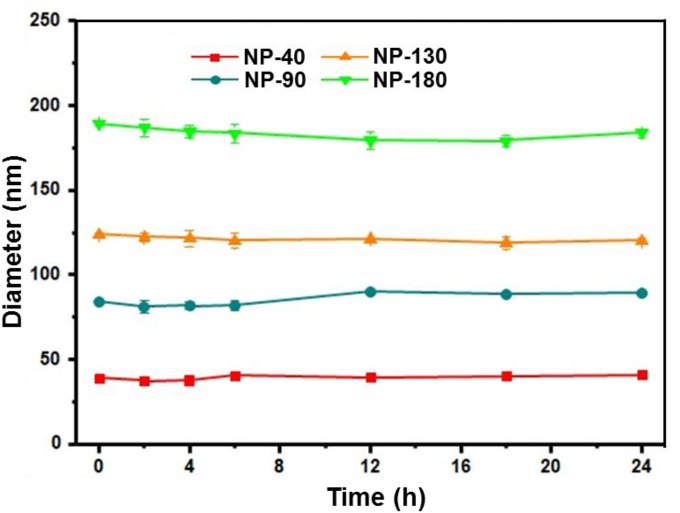
Size of the complexes formed between si*CCL-18* and NP-40, NP-90, NP-130, or NP-180 incubated in DMEM medium containing 10% FBS for different times.

**FIGURE 5:**
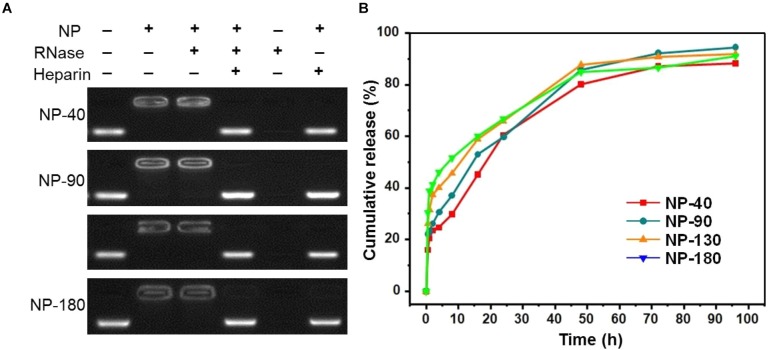
**(A)** Gel retardation assay of the siRNA loading capacity of the NP-40, NP-90, NP-130, and NP-180; **(B)** Cumulative siRNA release profile of the complexes formed between Cy5-si*CCL-18* and the NP-40, NP-90, NP-130, or NP-180.

We next used gel retardation assay to evaluate the siRNA loading capacity of the NPs. As shown in Figure 3, due to the presence of cationic PPEEA segment on the surface, all the NPs can effectively load si*CCL18* at an N/P ratio of 10:1, without apparent size change after si*CCL18* loading. Furthermore, all the si*CCL18* loaded NPs show good stability in 10% FBS-containing cell culture medium (Figure 4). More importantly, in comparison with the naked s*iCCL18*, the use of the NPs can protect the si*CCL18* from degradation by RNase. As shown in Figure 5A, with the protection by the NPs, the addition of RNase does not induce the siRNA degradation and the loaded siRNA can still bind with the NPs even under electric field. In contrast, without the NP protection, the naked siRNA has been degraded by the RNase and thus no siRNA band can be observed in the gel retardation assay experiment. All these results demonstrate the NP platform developed in this work shows a strong ability to load and protect the si*CCL-18*, which would thus ensure its bioactivity when used for regulation of macrophage activities. Notably, since we only varied the feed amount of hydrophobic PCL homopolymer to adjust the NP size, while the other two polymers (Meo-PEG-*b*-PCL and PCL-*b*-PPEEA) remain constant in the NP formulations, all the NPs showed the similar ability to load and release the siRNA (Figure 5B).

### Evaluation of CCL-18 Silencing

**FIGURE 6:**
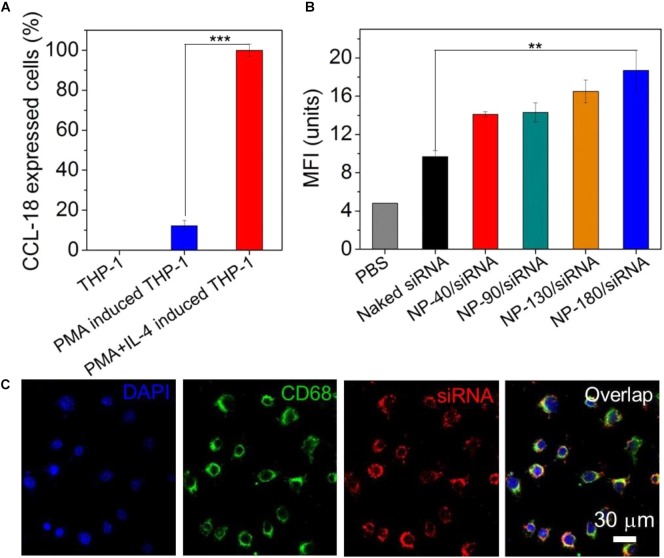
**(A)** The mRNA level of CCL-18 in the THP-1 cells treated with PMA and IL-4. **(B)** MFI determined by FACS analysis of the THP-1-originated macrophages incubated with the Cy5-si*CCL-18* loaded NPs for 4 h at a siRNA concentration of 200 nM. **(C)** Fluorescent images of the macrophages incubated with the Cy5-si*CCL-18* loaded NP-180 for 4 h at a siRNA concentration of 200 nM. The nuclei and macrophages were staining with DAPI as blue fluorescence and anti-CD68 as green fluorescence, respectively. ^∗∗^*P* < 0.01; ^∗∗∗^*P* < 0.001.

**FIGURE 7:**
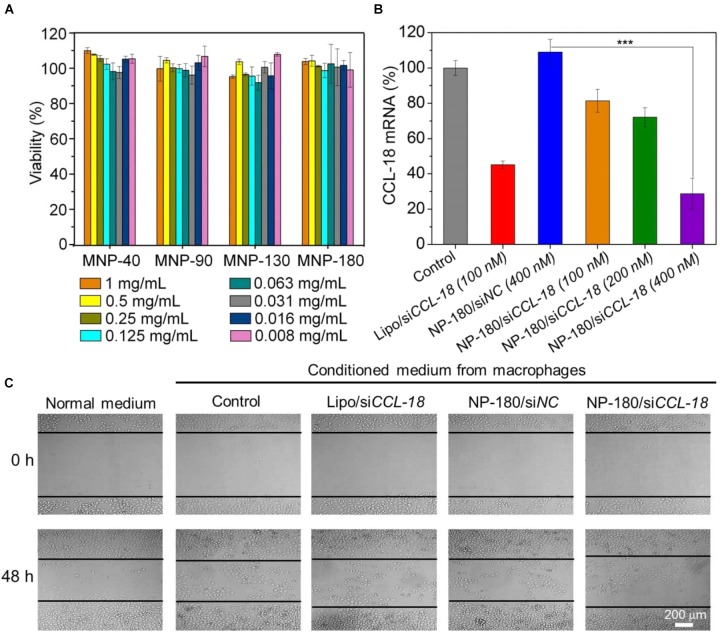
**(A)** Cell viability of the macrophages incubated with the si*CCL-18* loaded NPs at various concentrations for 48 h. **(B)** The mRNA level of CCL-18 in the macrophages treated by Lipo/si*CCL-18* complexes (Lipo/si*CCL-18*), negative control siRNA loaded NP-180 (NP-180/si*NC*), and si*CCL-18* loaded NP-180 (NP-180/si*CCL-18*). **(C)** Optical images of MDA-MB-231 cells incubated with the conditioned medium from the macrophages that treated with Lipo/si*CCL-18*, NP-180/si*NC*, and NP-180/si*CCL-18* at a siRNA concentration of 400 nM. ^∗∗∗^*P* < 0.001.

After validation of the siRNA loading ability of the NPs, we next examined whether these NPs can deliver siRNA to macrophages for gene silencing. THP-1, a human monocytic leukemic cell line, was used to construct M2-type macrophage-like cells through treatment with PMA and interleukin-4 (IL-4) (Liang et al., 2015). As shown in Figure 6A, the high expression of CCL-18, a well-known chemokine generated by M2-type macrophages (Chen et al., 2011), demonstrated the success of THP-1 cells differentiation from monocytes to M2-type macrophages (denoted THP-1-originated macrophages). With this encouraging result, we subsequently encapsulated dye-labeled si*CCL-18* (Cy5-si*CCL-18*) into the NPs and investigated their cellular uptake by the differentiated THP-1 macrophages obtained above. From the flow cytometry (FACS) analysis shown in Figure 6B, the THP-1-originated macrophages show higher uptake of the Cy5-si*CCL18* loaded NPs compared to the naked siRNA. The intracellular mean fluorescence intensity (MFI) is at least 1.3-fold stronger than that of the macrophages treated with the naked siRNA. Among these NP formulations, the uptake of NP-180 is highest and the possible reason is that macrophages are apt to internalize foreign materials with large size (Tabata and Ikada, 1988; Champion et al., 2008). Figure 6C shows the fluorescent images of macrophages incubated with the Cy5-si*CCL-18* loaded NP-180. Similar as the results of FACS analysis, the THP-1-originated macrophages show strong ability to internalize the siRNA loaded NP-180 as seen with bright red fluorescence and these NPs are mainly dispersed in the cytoplasm where siRNA functions (Whitehead et al., 2009). Although the THP-1-originated macrophages show higher cellular uptake of the NP-180, these NPs do not induce apparent cytotoxicity (Figure 7A).

Based on the results of FACS analysis and toxicity assay, the NP-180 shows higher uptake by the THP-1-originated macrophages with negligible toxicity. Therefore, we chose this NP platform as si*CCL-18* delivery tool to examine its gene silencing efficacy in macrophages. As shown in Figure 7B, the NP-180 can indeed transport si*CCL-18* into the THP-1-originated macrophages and thereby down-regulate CCL-18 expression. Compared to the macrophages without any treatment (Control), the mRNA level of CCL-18 is down-regulated by 20% at a si*CCL-18* concentration of 100 nM and more than 70% of CCL-18 is suppressed at a si*CCL-18* concentration of 400 nM.

### Evaluation of the Inhibition of Migration

It is known that CCL-18 is an important factor secreted by TAMs that can enhance breast cancer metastasis and therefore reduce patient survival (Chen et al., 2011; Su et al., 2014). Previous reports demonstrate that CCL-18 released by TAMs in breast cancer promotes the invasiveness of cancer cells by triggering integrin clustering and enhancing their adherence to extracellular matrix and silencing the CCL-18 expression can inhibit the consistent activation of downstream signal pathways to prevent the migration of breast cancer (Chen et al., 2011). Therefore, we finally simulated tumor microenvironment *in vitro* by incubating breast cancer cell line (MDA-MB-231) with the medium that was obtained from THP-1-originated macrophage (denoted conditioned medium), and examined the influence of CCL-18 silencing on the behavior of MDA-MB-231 cells. As shown Figure 7C, due to presence of secreted CCL-18 by macrophages, the addition of conditioned medium to MDA-MB-231 cells can enhance their migration compared to the cells incubated in normal medium. In contrast, after using si*CCL-18* loaded NP-180 to suppress CCL-18 expression in macrophages, the migration of MDA-MB-231 cells is significantly inhibited when incubated with the conditioned medium. This result is consistent with our previous reports (Chen et al., 2011; Su et al., 2014; Nie et al., 2017), and highlights the importance of CCL-18 to the breast cancer cell migration.

## Conclusion

We have developed a robust RNAi NP platform for efficient gene silencing in M2-type macrophages. Through varying the percentage of hydrophobic PCL homopolymer in the NP formulation, we successfully constructed four types of NPs with different sizes and systemically evaluated their siRNA loading ability and gene silencing efficacy. Experimental results demonstrate that the NP platform with larger size (NP-180) shows higher cellular uptake and efficient CCL-18 silencing in macrophages, leading to efficient inhibition of the breast cancer cell migration. Notably, this NP platform may passively target the tumor tissues via the EPR effect. However, the NP size is very large (∼180 nm) and will affect the therapeutic effect if used for *in vivo* regulation of macrophage behaviors. We are currently optimizing the NP formulation and small size NPs will be developed in the future for *in vivo* regulation of macrophage behaviors and cancer treatment.

## Author Contributions

SL, HY, YY, and XX conceived and designed the experiments. SL, JZ, WW, QL, and JC performed the experiments. SL, PS, XX, HY, and YY analyzed the data and co-wrote the paper.

## Conflict of Interest Statement

The authors declare that the research was conducted in the absence of any commercial or financial relationships that could be construed as a potential conflict of interest.
